# Aluminum, a Friend or Foe of Higher Plants in Acid Soils

**DOI:** 10.3389/fpls.2017.01767

**Published:** 2017-10-12

**Authors:** Emanuel Bojórquez-Quintal, Camilo Escalante-Magaña, Ileana Echevarría-Machado, Manuel Martínez-Estévez

**Affiliations:** ^1^CONACYT-Laboratorio de Análisis y Diagnóstico del Patrimonio, El Colegio de Michoacán, La Piedad, Mexico; ^2^Unidad de Bioquímica y Biología Molecular de Plantas, Centro de Investigación Científica de Yucatán, Mérida, Mexico

**Keywords:** acid soils, aluminum, aluminum toxicity, beneficial effect of aluminum, mechanisms of tolerance, metal, plant growth stimulation

## Abstract

Aluminum (Al) is the most abundant metal in the earth’s crust, but its availability depends on soil pH. Despite this abundance, Al is not considered an essential element and so far no experimental evidence has been put forward for a biological role. In plants and other organisms, Al can have a beneficial or toxic effect, depending on factors such as, metal concentration, the chemical form of Al, growth conditions and plant species. Here we review recent advances in the study of Al in plants at physiological, biochemical and molecular levels, focusing mainly on the beneficial effect of Al in plants (stimulation of root growth, increased nutrient uptake, the increase in enzyme activity, and others). In addition, we discuss the possible mechanisms involved in improving the growth of plants cultivated in soils with acid pH, as well as mechanisms of tolerance to the toxic effect of Al.

## Introduction

**FIGURE 1:**
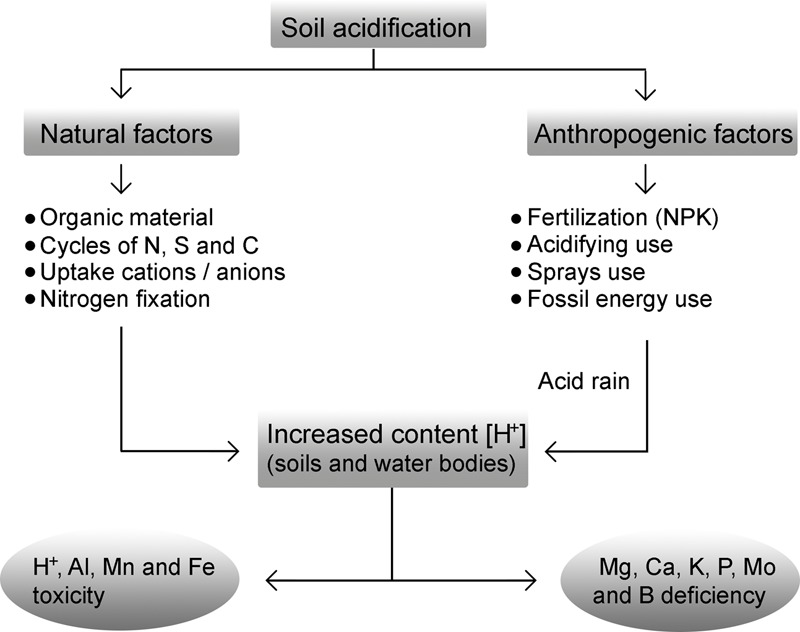
Soil acidification is a phenomenon determined by natural and anthropogenic factors. The decomposition of the organic matter, the imbalance of the N, S, and C cycles, the excess in the cation uptake on anions and the N fixation by the legumes influence the concentration of protons [H^+^] in the soil solution. Anthropogenic factors such as the use of fertilizers (nitrogen, phosphorus, and potassium, NPK), the use of acidifiers and aerosols (H_2_S, H_2_SO_4_, HF, and Cl_2_), and the emission of gasses (CO_2_, NO_2_, and SO_2_) into the atmosphere by use of fossil energy give rise to environmental pollution. Such molecules find their way into soil and water bodies in the form of acid rain, causing acidification of soils and the release of Al ions in a form easily absorbed by the plant root system, which is extremely toxic. Also, nutrient deficiency (P, Mg, and K) and toxicity by other metals (Mn and Fe) may occur.

Acid soils, also called ultisols or oxisols, have a pH of 5.5 or lower; they are widely distributed in tropical and subtropical regions, constituting approximately 30% of the total area of the planet and 50% of the arable land in the world, as well as providing between 25 and 80% of vegetable production (Sade et al., 2016). Soil acidification can occur due to natural and/or anthropogenic processes (**Figure 1**). Most acid soils occur in the tropics and subtropics, where acidification is a natural process. This situation can be worsened by environmental contamination through the use of fertilizers and acidifying substances, as well as the use of fossil energy sources such as coal and oil which release nitrogen dioxide (NO_2_) and sulfur dioxide (SO_2_) into the atmosphere and which, in the presence of oxidizing agents, give rise to nitric acid (HNO_3_) and sulfuric acid (H_2_SO_4_), thereby increasing the precipitation of acid rain and acidification of the bodies of water and the soil. Moreover, organic material in decomposition, imbalance in N, S, and C cycles, excessive uptake of cations over anions and the uptake of nitrogen by leguminous crops all increase the concentration of H^+^ and reduce soil pH (**Figure 1**). Acid soils are characterized by a deficiency in nutrients and toxicity by metals, such as manganese (Mn), iron (Fe) and Al; with toxicity by Al being the main factor limiting plant growth in acid soils (Kochian et al., 2004; Gupta et al., 2013; Bose et al., 2015).

**FIGURE 2:**
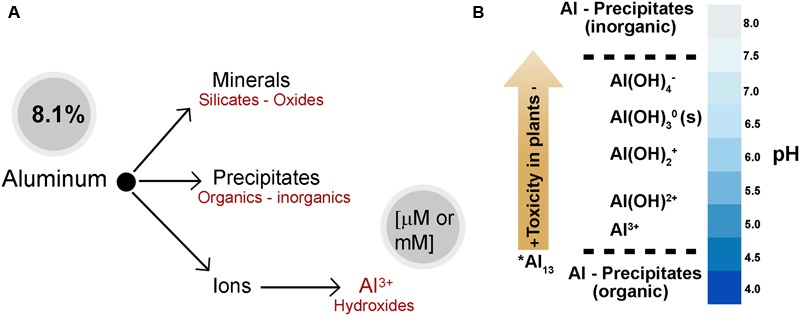
Aluminum abundance and speciation in the earth’s crust. **(A)** Different forms of Al in the soil and water. Aluminum is mainly found in the mineral form (aluminosilicates and aluminum oxides). In addition, Al can be found as precipitates or conjugated organic and inorganic, and molecular ions depending on the soil pH. **(B)** Al-speciation in soil solution. Al concentration and the speciation of Al depend on the pH and the chemical environment of the soil solution. However, a very toxic polynuclear Al species (^∗^Al_13_) depends on the total concentration of Al. Molecular aluminum (mononuclear) exists as hydroxyaluminum: Al/Al(H_2_O)_6_^3+^, AlOH^2+^, Al(OH)_2_^+^, Al(OH)_3_ y Al(OH)_4_^-^ Trivalent aluminum (Al) is the most abundant form and has the greatest impact on plant growth at pH < 5. At pH > 5–6, the dominant species are AlOH^2+^ and Al(OH)_2_^+^, which are not as toxic to plants as Al. When the pH is neutral, Al(OH)_3_ or gibbsite occurs; however, it is non-toxic and relatively insoluble. Aluminate, Al(OH)_4_^-^, is the dominant specie when the pH is alkaline (pH > 7). (Kinraide, 1991; Delhaize and Ryan, 1995; Brautigan et al., 2012; Hagvall et al., 2015; Kisnieriené and Lapeikaité, 2015).

Aluminum is the most abundant metal on earth and it is the third most abundant element (after oxygen and silicon) in the earth’s crust, representing approximately 8.1% of its content in weight (**Figure 2A**). Despite being ubiquitous and available during the life cycle of plants, Al has no specific biological function (Poschenrieder et al., 2008). Organisms are not usually exposed to relevant concentrations of Al in the soil as it is mainly found in the form of a mineral (aluminosilicates and aluminum oxides); however, in aqueous solutions and at different pH, Al hydrolyzes water molecules to form aluminum hydroxide (**Figure 2A**). Total Al concentration in the soil and the speciation of Al depend on the pH and the chemical environment of the solution (Kisnieriené and Lapeikaité, 2015). The toxic effect of the different forms of Al (speciation) on plant growth diminishes in the following order: Al, Al(OH)^2+^, Al(OH)_2_^+^, Al(OH)_4_^-^ (**Figure 2B**). At a low pH (about 4.3) trivalent aluminum (Al^3+^) is the most abundant form and has the greatest impact on plant growth. In contrast, precipitated or chelated Al with organic compounds is not toxic for plants (Nogueirol et al., 2015).

It was recognized for the first time, over 100 years ago, that concentrations of soluble Al increase in acid soils (Veitch, 1904) and that this soluble Al is toxic for plant growth, the main effect of Al toxicity being inhibition of root growth (Daikuhara, 1914; Miyake, 1916; Magistad, 1925; Kopittke et al., 2016). Surprisingly, stimulation of root growth is one of the beneficial effects of Al. The impact of Al on plant growth, both toxic and beneficial, depends on the concentration of the metal and varies according to the plant species, which includes the genotype within the same species, physiological age, growth conditions and the duration of exposure to the metal. According to Barcelo and Poschenrieder (2002) and Zhou et al. (2011), with respect to root growth, three responses can be observed depending on the concentration of Al. (1) Root growth is not affected at low concentrations of Al; however, at a higher concentration it is diminished; (2) root growth is stimulated at low concentrations of Al, but it is affected at high concentrations; (3) inhibition of root growth at low concentrations or short time periods, but little or no effect at high concentrations or long time periods. A fourth response is presented in genotypes where root growth is not affected even at very high concentrations of Al, indicating that different plant species differ in their response mechanisms to stress by Al at cellular and tissue levels, as well as whole-plant level.

## Beneficial Effect of Aluminum in Plants

**FIGURE 3:**
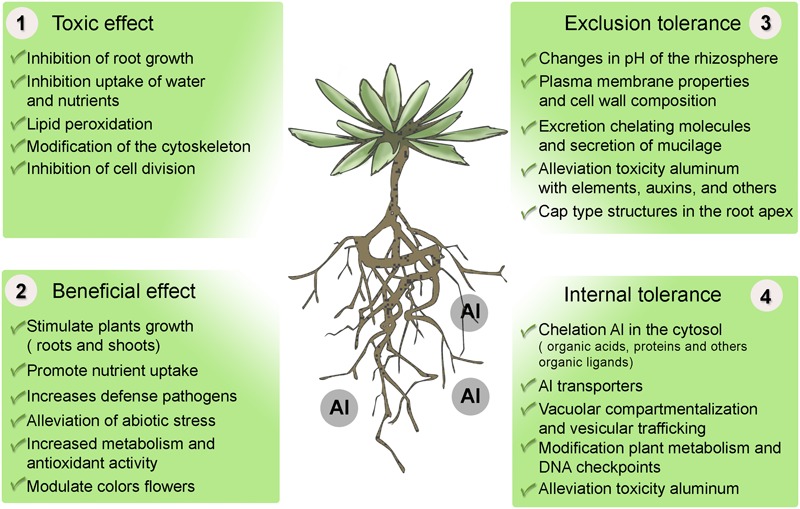
Effect of aluminum on plants and mechanisms of tolerance to stress by aluminum. (1) Toxicity of Al in plants. (2) Beneficial effect of Al in some taxas, mainly species adapted to acid soils. (3) Mechanisms of exclusion, resistance or alleviation of Al uptake, and (4) Mechanisms of internal tolerance to stress by Al in plants.

Since Maze (1915) and Stoklasa (1922) reported, for the first time, on the possible role of Al in plant growth and development, considerable interest has been shown in studies on the beneficial effect of Al on plants. In recent years, an increasing number of articles dealing with this topic have been published. However, to date, no evidence has been put forward as to the essentiality of this metal. Although the exact mechanism causing the beneficial effect of Al is still unknown, a few possible mechanisms have been suggested to explain it (**Figure 3**).

### Plant Growth Stimulation by Aluminum

Growth stimulation induced by Al has been observed frequently in native plants, or plants which have adapted to acid soils when Al is mainly administered at low concentrations (Yoshii, 1937; Osaki et al., 1997; Pilon-Smits et al., 2009). In the case of the *Tabebuia chrysantha* tree, low levels of Al stimulated the synthesis of root biomass; in contrast, a high level of Al had an inhibitory effect (Rehmus et al., 2014). According to Poschenrieder et al. (2015) two patterns of growth stimulation resulting from the administration of Al can be observed in plants: a transitory increase (short term) in growth, observed mainly in laboratory studies, and a permanent increase in productivity induced by Al in the highly tolerant plants. For example, prevention of H^+^ toxicity and an increase in root elongation induced by Al is a transitory effect which occurs for short periods (Kinraide et al., 1992; Llugany et al., 1995; Kinraide, 1998). However, in an *in vitro* culture of coffee (*Coffea arabica*) seedlings, growth stimulation of the primary root induced by Al occurred from day 10 to day 30 of culture (Bojórquez-Quintal et al., 2014). In *Betula pendula* and *Quercus serrata* trees, growth increase (roots and leaves) induced by Al has been observed in the long term, even after 28 days and 17 months, respectively (Kidd and Proctor, 2000; Tomioka and Takenaka, 2007). Similar results were reported in *Conostegia xalapensis* after 24 days of treatment with 0.5 and 1 mM of Al. The treatment with Al increased root biomass and the number of lateral roots. *C. xalapensis* is a shrub, hyperaccumulator of Al, which is common to Mexico and Central America, and colonizes pollution-perturbed areas. A possible role as an indicator species for toxicity and contamination of Al has been suggested for this species (González-Santana et al., 2012).

*Camellia sinensis, Miscanthus sinensis, Quercus serrata*, and *Melastoma malabathricum* are tropical species, hyperaccumulators of Al, which grow in acid soils (Yoshii, 1937; Osaki et al., 1997; Ghanati et al., 2005; Tomioka et al., 2005). In tea (*C. sinensis*) and *Q. serrata*, it is well known that Al potentializes biomass growth, root elongation and both short and long term proliferation of lateral roots (Ghanati et al., 2005; Tomioka et al., 2005; Mukhopadyay et al., 2012; Hajiboland et al., 2013b; Xu et al., 2016). In the case of tea, it has been demonstrated that root growth is stimulated in the presence of Al, while in the absence of the metal, growth of the root and the plant is delayed (Tsuji et al., 1994; Fung et al., 2008). In *M. malabathricum* it has been suggested that Al is essential for the growth of this plant; in the absence of the metal, chlorosis, morphological changes and leaf curling have been observed (Watanabe et al., 2006). The Melastoma plant secretes mucilage in the roots in order to accumulate Al in soils with poor availability of this element and the accumulation of Al increases the growth of roots and shoots (Watanabe et al., 2005, 2008a,b). Morphological changes in the root, such as thickening, white coloration and elongation have also been reported in *C. sinensis, Q. acutissima, Cinnamomum camphora, Eucalyptus viminalis, Q. serrata, M. malabathricum*, and *Symplocos paniculata* in the presence of Al (Oda, 2003; Ghanati et al., 2005; Watanabe et al., 2006; Schmitt et al., 2016a).

Despite the fact that the beneficial effect of Al in plants has been reported mainly in woody species adapted to acid soils (Osaki et al., 1997; Hajiboland et al., 2013b), there are reports available on species of economic importance, such as rice (*Oryza sativa*) and corn (*Zea mays*) (Watanabe et al., 1997; Famoso et al., 2011; Wang et al., 2015b). In rice varieties tolerant to Al, an increase in root growth has been observed in the presence of 160 and 200 μM of Al (Famoso et al., 2011; Moreno-Alvarado et al., 2017). The administration of a low dosage of Al in corn inhibited root growth, but increased leaf growth (Wang et al., 2015b). Similarly, an increase in the Al content of horticultural species, such as turnip (*Brassica rapa*) and the legume *Glycine max* increases elongation and activity of the root (Rufty et al., 1995; Yu et al., 2011).

Aluminum can also stimulate the growth of other organisms. In marine environments, Al increases the growth and biomass accumulation of phytoplankton, mainly diatoms (Saçan et al., 2007; Golding et al., 2015; Zhou L. et al., 2016). In a mutant of *Saccharomyces cerevisiae* (*zrt1*Δ), deficient in zinc uptake, the addition of Al restored growth to a level comparable to that of the wild strain and promoted the uptake of Zn (Tamura and Yoshimura, 2008). In microorganisms such as *Frankia* (a nitrogen-fixing bacteria), an increase in growth (*in vitro*) was observed with the addition of 500 μM of Al; similarly, it was possible to observe that Al prevented the inhibitory effect of the acidic pH (Igual and Dawson, 1999). Aluminum is also involved in bone formation in animal cells (Quarles et al., 1988, 1989). It has been observed that the addition of Al increases the proliferation and differentiation of human and chicken osteoblasts (Lau et al., 1991). Moreover, it has been demonstrated that Al induces the synthesis of DNA in osteoblasts (Quarles et al., 1994) and acts as mitogen in epithelial cells of mice (Jones et al., 1986).

### Aluminum Promotes Nutrient Uptake

One of the possible reasons explaining the stimulation of plant growth induced by Al is the promotion of nutrient uptake. In hyperaccumulator plants, Al can stimulate or have no effect on essential nutrient uptake (Malta et al., 2016). In different plant species, nitrogen (N), phosphorous (P), and potassium (K) uptake has been considered the mechanism responsible for the stimulation of root growth induced by Al (Osaki et al., 1997). In tea plants, the stimulation of root growth has been explained as a consequence of an increased uptake of some macronutrients (Fung et al., 2008). In *M. malabathricum, Q. serrata* and tea the uptake and accumulation of P in the roots and leaves of the plants increased in the presence of Al. It has been suggested that the stimulation of root growth and the increase in P could be due to precipitation of the Al-P complex on the root surface and/or in the Donnan free space (apoplast) and in some way the plants use the precipitated P (Konishi, 1992; Osaki et al., 1997; Tomioka et al., 2005). Similarly, Al stimulates alkaline phosphatase activity and organic P uptake in the marine diatom *Thalassiosira weissflogii* (Zhou L. et al., 2016).

In *Q. serrata*, stimulation of root growth was associated with the activation of the nitrate reductase and the increase in NO_3_^-^ uptake (Tomioka et al., 2007, 2012). In species of the genus *Symplocos* there is a positive correlation between the calcium (Ca) uptake and the level of Al (Schmitt et al., 2016a). Similarly, in *C. arabica* roots an increase in the content of K and Ca was observed with the concentration of Al which stimulated primary root growth (Bojórquez-Quintal et al., 2014). All the data presented suggest that Al can induce the expression or activity of transport proteins (channels and transporters) and change the membrane potential and proton flux (H^+^) which promotes the fluxes of nutrients in the plants. There is evidence in wheat roots (*Triticum aestivum*) that Al increased the extrusion of H^+^ and the polarity of the membrane, which could be associated with a greater nutrient uptake and growth increase (Kinraide et al., 1992; Kinraide, 1993). Moreover, in *Arabidopsis thaliana*, Al induces depolarization of the membrane, K and H^+^ influx, and reduces K efflux (Bose et al., 2010a,b). Magnesium (Mg) can prevent the toxic effect of Al on plants (Bose et al., 2011). In a mutant of *Arabidopsis* (*alr-140*), an increase in influx and intracellular content of Mg induced by Al has been observed; it has also been suggested that Al can activate channels and Mg transporters in Al-resistant plants (Bose et al., 2013).

### Aluminum Prevents Biotic and Abiotic Stress

The beneficial elements, including Al, can increase tolerance to abiotic stress (ion toxicity and nutrient deficiency) and resistance to biotic stress (herbivores and pathogens) (Kaur et al., 2016). Pilon-Smits et al. (2009) mention that plants which are hyperaccumulators of Al (1 g Al kg^-1^ dry weight) can use this metal in their tissues to discourage herbivores, as was observed in the application of Al to prevent herbivory in *Festuca arundinacea* (Potter et al., 1996). Due to the fact that Al can be toxic for some pathogenic microorganisms, a number of salts containing Al have been used to control diseases caused by fungi in crops of carrot (*Daucus carota*) and potato (*Solanum tuberosum*) (Kolaei et al., 2013). In *S. tuberosum*, treatment with Al increased resistance to the oomycete *Phytophthora infestans* and to the pathogenic fungus *Thielaviopsis basicola* Ferraris. The addition of Al inhibited the germination of spores and fungus growth (Meyer et al., 1994; Andrivon, 1995). The protective capacity of Al against *P. infestans* was associated with the accumulation of H_2_O_2_ in the roots and the activation of the acquired systemic response depending on salicylic acid and nitric oxide (Arasimowicz-Jelonek et al., 2014).

Aluminum can prevent the effects of H^+^ toxicity and that of different elements when found in excess. The increase in root biomass of tree species after treatment with Al has been associated with the lessening of H^+^ toxicity (Thornton et al., 1986, 1989). This mechanism has also been suggested in wheat, Japanese radish (*Raphanus sativus* var. Longipinnatus) and pea plants (*Pisum sativum*), but not in *Q. serrata* (Kinraide et al., 1992; Kinraide, 1993; Tomioka et al., 2005). In acid soils, the availability of Al, iron (Fe) and manganese (Mn) is high; thus, the plants growing in these soils can present toxicity of these metals (**Figure 1**). An excess of Fe induces the production of reactive oxygen species (ROS), leading to the interruption of several cellular functions. In plants of *M. malabathricum* and *C. sinensis*, growth stimulation is accompanied by the prevention of iron toxicity. In these species, Al prevents bronzing of the leaves due to the toxic effect of Fe and also reduces the Fe content in leaves and roots (Watanabe et al., 2006; Watanabe and Osaki, 2009; Hajiboland et al., 2013a).

In some plant species, Al can prevent the Mn toxicity (Yang et al., 2009; Wang et al., 2015e). In rice, the prevention of Mn toxicity induced by Al can be attributed to the reduced metal accumulation in the shoots as a result of the decrease in Mn uptake in the roots. The reduction of Mn uptake in the roots was a consequence of changes in the membrane potential. In addition, Al brought about an increase in insoluble Mn in the root and changed the properties of junction to the cell wall, making Mn less available in the rice roots (Wang et al., 2015e). Aluminum can also help to detoxify fluoride (F) in tea plants by forming Al-F compounds. In the absence of the metal, the tea plants were sensitive to F (Yang Y. et al., 2016). Aluminum is also able to prevent toxicity caused by other elements, particularly P, zinc (Zn) and copper (Cu) (Asher, 1991). Also, Al improves plant growth under nutrient deficiency; for example, supplementation with Al enhanced the growth of tea plants deficient in boron (B). Under B deficiency conditions, Al was able to positively regulate the metabolism of N and carbon (C) and antioxidant defense activity, while increasing the uptake and transportation of B in tea plants (Hajiboland et al., 2014, 2015). Under limiting conditions of P, Al stimulates P uptake in microalgae (Zhou L. et al., 2016).

### Beneficial Effects of Aluminum on Plant Metabolism

Investigation on the beneficial effect of Al has focused mainly on physiological aspects such as the promotion of nutrient uptake; however, recent advances have suggested the possible role of Al in plant metabolism (Xu et al., 2016). In tea plants, Al is retained in the apoplast of leaf epidermal cells as a mechanism of Al-resistance (Tolrà et al., 2011). In the aerial part of the plant, the stimulatory effect of Al has been attributed to the increase in photosynthesis and the activation of antioxidant defense (Hajiboland et al., 2013b). Similarly, in roots and cell suspensions of tea plants, Al induces the activation of antioxidant enzymes, increases the integrity of the membrane and reduces lignification and aging, which could be a possible reason for the stimulation of root growth (Ghanati et al., 2005). In *C. xalapensis* plants treated with Al, the increase in biomass and in the number of lateral roots coincided with greater activity of the glutathione reductase and superoxide dismutase, at low levels of ROS (González-Santana et al., 2012). In *Q. serrata*, stimulation of root growth by Al is associated with an increase in the activation of nitrate reductase and the photosynthetic rate (Tomioka and Takenaka, 2007; Tomioka et al., 2007, 2012; Moriyama et al., 2016).

The beneficial effect of Al on plants has also been associated with the regulation of C and N metabolism. The shrub *M. malabathricum* changes the metabolism of organic acids (OA) in the absence or presence of Al; more specifically, it increases the synthesis of citrate and diminishes that of malate (Watanabe and Osaki, 2001), suggesting the possible role of Al in the regulation of the expression and activation of genes and proteins associated with the biosynthesis of OA with respect to growth stimulation. In *M. malabathricum*, citrate is necessary for the transportation of Al from the roots to aerial part of the plant (Watanabe et al., 2005). In tea plant roots, it has been suggested that the secretion of caffeine induced by Al can increase root growth by inhibiting callus deposits (Morita et al., 2011). Also, Al positively regulates the biosynthetic pathway of caffeine in suspension cells of *C. arabica*. This regulation may be accomplished by activation of a signal transduction pathway through Ca and ROS (Pech-Ku et al., 2017). There is also evidence indicating that Al increases the content of chlorophyll, carotenoids, sugars, amin oacids such as proline and cysteine, hormones, and metabolites of the Shikimico acid pathway in woody and crop plants (Hajiboland et al., 2013b; Moriyama et al., 2016; Xu et al., 2016; Moreno-Alvarado et al., 2017). In fact, glucose has been suggested as an energy source which is a key element in the promotion of root growth, in the presence of Al. Moreover, glucose and abscisic acid (ABA) may participate as signaling molecules which promote the root growth induced by Al, acting on the metabolism of N and C (Moriyama et al., 2016). For instance, Al increases the concentration of soluble sugars and differentially regulates the expression of NAC transcription factors, which in turn may enhance growth and biomass production in rice plants (Moreno-Alvarado et al., 2017).

In corn, stimulation of leaf growth has been associated with greater protein synthesis (Wang et al., 2015b). In coffee seedlings, the activity of phospholipase C, an enzyme which participates in signal transduction, was increased in the concentration of Al where it was possible to observe stimulation and inhibition of root growth, suggesting a possible participation in the signal-transduction pathway mediated by inositol phosphate in the beneficial and toxic effect of Al in plants (Bojórquez-Quintal et al., 2014). It has also been suggested that the beneficial effect of Al on root growth and morphological changes is associated with changes in the levels of plant growth regulators (PGR). Aluminum is capable of inducing, directly or indirectly, synthesis or transportation of PGR. In the conifer *Picea abies*, an increase in the levels of indole-acetic acid (IAA), cytokinins and gibberellins has been reported in the roots in the presence of Al; these changes are in correlation with the morphological changes and the stimulation of lateral roots close to the root apex. The formation of new roots in *Q. acutissima, C. camphora*, and *Q. serrata* was accelerated by the Al treatment and correlated with low concentrations of IAA and an increase in the concentration of cytokinins and ABA in the roots (Oda, 2003; Moriyama et al., 2016).

### Aluminum Modulates Floral Colors

Aluminum plays an important role in the color changes of some hyperaccumulator plants. In hydrangea (*Hydrangea macrophylla*), Al and pH are important factors for the change of color in the sepals, from red to blue (Ito et al., 2009; Schreiber et al., 2011). The blue coloring is formed from the union of Al with metalloanthocyanins, delphinidin 3-glucoside and 5-*O*-caffeoylquinic acid (Oyama et al., 2015; Kodama et al., 2016). In the genus *Camellia*, the purple color of *Camellia japonica* flowers (cv. Sennen-fujimurasaki) and the intense yellow tone of *Camellia chrysanthaes* are generated by the chelation of the Al ions with anthocyanins and flavonoids (quercetin), respectively (Tanikawa et al., 2008, 2016). Anthropologically, the leaves and bark of species of the genus *Simplocos* are used by weavers as mordant in textile dyeing due to their high content of Al (Schmitt et al., 2016b).

## Aluminum Toxicity

Aluminum is also the primary factor in reducing crop yields in acid soils (Ma et al., 2002; Kochian et al., 2005). The initial and most dramatic Al toxicity symptom is the inhibition of root elongation (Delhaize and Ryan, 1995). Because Al is a highly reactive element, there are innumerable mechanisms of toxicity involving the cell wall (Jones et al., 2006; Zhang et al., 2007) and plasma membrane, where it can modify its structure, as well as the nearby ionic medium to wall, both disturb the transport of ions and cause an improper balance of nutrients (Bose et al., 2011). Also, Al can affect the constituents’ symplast (calmodulin) (Tokizawa et al., 2015), apoplast (pectin matrix) (Eticha et al., 2005; Delhaize et al., 2007) and DNA in cells of plant roots (Kochian et al., 2004; Sade et al., 2016).

Aluminum is widely reported as toxic to most plants (Sade et al., 2016). There are a number of symptoms caused by aluminum toxicity in plants (**Figure 3**), all associated with severe changes in the root system (Kopittke and Blamey, 2016). Al interferes with cell division at the root apex and lateral roots, increases the rigidity of the cell wall by cross-linking of pectins and reduces DNA replication because of increased rigidity of the double helix (Zhang et al., 2014; Eekhout et al., 2017). Moreover, Al induces a series of cellular toxic changes concerning cell division and nucleolus, and localization and expression of the nucleolar proteins such as fibrillarin (Zhang et al., 2014). Aluminum tends to bond with phosphorus (P) in a less available and insoluble form in soils and plant roots, thereby creating a P deficiency for plant growth. Aluminum also decreases root respiration, interferes with enzymes governing the deposition of polysaccharides in cell walls, decreases the synthesis and transport of cytokinins, and modifies the structure and function of plasma membranes which interfere with the uptake, transport, and use of multiple elements (Ca, Mg, P, and K), as well as water uptake by roots plants (Foy, 1974; Foy et al., 1978; Kochian et al., 2004).

In plants these symptoms are linked to disorders that are generally divided into two categories: (1) long-term responses, requiring hours to develop, and (2) short-term responses that can be measured within a few minutes or even a few seconds after exposure to Al (Taylor, 1988; Simoes et al., 2012). The first signs of these responses related to Al toxicity have been observed after one hour (Ownby and Popham, 1989). Moreover, the most important response to the application of Al seems to be short term interruption of Ca influx through the plasma membrane (Jones et al., 1998; Panda and Matsumoto, 2007).

Aluminum and other metals are non-biodegradable, they remain in the environment and are able to circulate in the food chain, posing a serious threat not only to plants but also to animals and humans (Jackson and Huang, 1983; Exley, 2003; Ashenef, 2014). For example, tea plants contain a substantial amount of Al in leaves, and thus it is present in tea leaf infusions. Although only a small proportion of Al is available for absorption in the gastrointestinal tract and the renal excretion of Al is fairly effective, this metal can cause serious problems or possible health risks in humans (Exley, 2003; Mehra and Baker, 2007). High Al content in the human body has been hypothesized as having possible links with various diseases, such as encephalopathy, dementia, Alzheimer’s disease, osteomalacia fractures and high levels of bone Al; therefore tea-drinkers should be forewarned of the risks (Anitha and Rao, 2002; Mehra and Baker, 2007; Ashenef, 2014).

## Mechanisms of Tolerance to Aluminum

Plants have developed different mechanisms of tolerance to counteract the toxic effects of Al. These mechanisms can be divided into two forms (**Figure 3**): mechanisms of exclusion or resistance to Al, the function being to avoid or reduce the entrance of Al to the cell; and mechanisms of internal tolerance which compartmentalize Al in vacuoles or stabilize them in order to inhibit its toxicity.

### Tolerance to Aluminum – Mechanisms of Exclusion

#### Changes in the Rhizosphere pH

A small increment in the rhizospheres pH reduces the solubility, activity, toxicity and content of Al in plants through exclusion of the metal in the root apoplast (Yang Y. et al., 2011). The *Arabidopsis alr-104* mutant Al-resistant and other plant species increase the apoplastic pH through the H^+^ and NH_4_^+^ influx and the efflux of OA in the root apex, in the presence of Al (Foy et al., 1965; Degenhardt et al., 1998; Larsen et al., 1998; Houman et al., 2009; Bose et al., 2010b; Wang et al., 2015a). In squash (*Cucurbita pepo*) and wheat, changes in rhizosphere pH and resistance to stress due to Al are regulated by H^+^-ATPasa of the plasma membrane (Ahn et al., 2001, 2002; Yang Y. et al., 2011). OA can also increase or reduce rhizosphere pH in the presence of other metals, as well as Al (Kochian et al., 2004; Zeng et al., 2008; Javed et al., 2013). In the case of tea, the Al uptake induces H^+^ efflux from the roots, suggesting a mechanism of soil acidification (Wan et al., 2012). Aluminum plays a positive role in growth increase in tea (Konishi et al., 1985), particularly in the promotion of root elongation. Soil acidification might maintain the solubility and Al uptake in tea. In contrast, acidification of the rhizosphere increases toxicity and metal accumulation in Al-sensitive species (Houman et al., 2009).

#### Changes in Cell Wall Composition and Plasma Membrane Properties

The root cell wall is the main binding site of Al and thus is the target of Al toxicity and exclusion in plants (Horst et al., 2010; Yang J.L. et al., 2011; Kopittke et al., 2015; Liu S. et al., 2016). At present, physiological, biochemical and molecular evidence is available which has demonstrated that modification of the cell wall composition plays an important role in the resistance to Al (Levesque-Tremblay et al., 2015; Che et al., 2016; Zhang et al., 2016). The increase in the polysaccharide content of the cell wall induced by Al can reduce water and nutrient uptake, as well as cellular wall elasticity (Nguyen et al., 2005). Among the cereals, rice is the crop with most tolerance to Al (Yang et al., 2008; Famoso et al., 2010). In rice cultivars which differ in their Al-resistance, a positive correlation between polysaccharide content (pectin and hemicellulose) in the root apex and the accumulation of Al has been observed, indicating the importance of the cell wall composition in the exclusion of Al (Yang et al., 2008). The pectin content and its degree of methylation in the cell wall contribute to the differences in resistance to Al (Eticha et al., 2005; Yang J.L. et al., 2011). In *Arabidopsis* and other plant species, the regulation of genes and expansin enzymes, pectin methyltransferases and xiloglucano endohydrolases reduce binding and accumulation of Al in the root apex by changing the load and the porosity of the cell wall (Yang J.L. et al., 2011; Zhu et al., 2014; Zhang et al., 2016). The transporter ABC formed by the complex STAR1/STAR2 transport UDP-glucose and it can modify the cell wall under stress by Al (Huang et al., 2009).

The physicochemical and physiological properties of the plasma membrane also affect tolerance of the plants to Al. The lipid composition of the plasma membrane (PM) has an important role in Al-tolerance (Wagatsuma et al., 2015). The phospholipids create a negative charge on the surface of the PM and increase the sensitivity to Al, as a result of the union of the metal to the PM. In rice and timber species (*M. malabathricum* and *M. cajuputi*), Al-tolerance increased in response to a decrease in the proportion of phospholipids of the root cell PM (Maejima and Watanabe, 2014). Similarly, the sterol content also plays an important role in the tolerance to stress by Al. High sterol content in combination with low contents of phospholipids is a common strategy for Al-tolerance in different plant species (Khan et al., 2009; Wagatsuma et al., 2015). It has also been suggested that a small peptide, anchoring the PM, could prevent Al-influx in root cells through bonding with cation and thus contribute to the resistance in rice (Xia et al., 2013).

#### Excretion of Chelating Molecules and Mucilage Secretion

The release of OA in the root is the Al exclusion mechanism most widely described in plants (Kochian et al., 2015), with a wide natural variation in different crops, mainly in cereals (Brunner and Sperisen, 2013; Schroeder et al., 2013). The organic anions, malate, citrate and oxalate are secreted by the root and chelate Al in a non-toxic Al-OA complex, protecting the root apex and permitting it to grow. Malate and citrate are ubiquitous in all plant cells given that they are part of the tricarboxylic acid (TCA) cycle in the mitochondria, while oxalate participates in the regulation of Ca and the detoxification of metals (Brunner and Sperisen, 2013). Evidence in different species of cultivable plants and timber trees indicates that OA efflux confers resistance to Al (Delhaize et al., 1993; Brunner and Sperisen, 2013; Yang et al., 2013).

In a wheat genotype resistant to Al (Delhaize et al., 1993), the first gene of a malate transporter activated by Al (*TaALMT1*) was cloned, which codifies an anionic channel activated by Al that facilitates the malate efflux from the root. *TaALMT1* was the first gene of resistance to Al cloned in plants (Sasaki et al., 2004). To date, several *ALMT1* type genes have been isolated in different plant species (*AtALMT1*, Hoekenga et al., 2006; *BnALMT1* and *BnALMT2*, Ligaba et al., 2006; *HvALMT1*, Gruber et al., 2010; *ZmALMT2*, Ligaba et al., 2012). The heterologous expression of *TaALMT1* in barley (*Hordeum vulgare*) and tobacco (*Nicotiana tabacum*) allowed the malate efflux and an increase in Al-resistance (Delhaize et al., 2004; Zhang et al., 2008). On the other hand, the citrate efflux is mediated by the family of MATE proteins (Multidrug and toxic compound extrusion). *MATE* genes have been cloned and identified in *Arabidopsis* (*AtMATE*, Liu et al., 2009), sorghum (*SbMATE*, Magalhaes et al., 2007), barley (*HvAACT1*-*HvMATE1*, Furukawa et al., 2007), wheat (*TaMATE1*, García-Oliveira et al., 2014), poplar (*PtMATE1*, Grisel et al., 2010), rice (*OsFRD1*; Yokosho et al., 2011), rice bean (*VuMATE1*, Yang X.Y. et al., 2011), corn (*ZmMATE1-ZmMATE2*, Maron et al., 2010, 2013) and soybean (*GmMATE1-GmMATE117*, Liu J. et al., 2016). The plasma membrane H^+^-ATPase plays an important role in OA exudation in the root by the transporters ALMT and MATE (Yu W. et al., 2016); however, in cluster roots of white lupin (*Lupinus albus*), the citrate exudation induced by combined treatment with P-deficiency and Al is independent of H^+^-ATPase PM activity and dependent on K efflux (Zeng H. et al., 2013). To date, the molecular components of oxalate efflux have not been identified. In tea plants and other woody species such as poplar (*Populus tremula*) and buckwheat (*Fagopyrum esculentum*), oxalate is an important element in the detoxification of Al in the root (Qin et al., 2007; Morita et al., 2008; Wang et al., 2015a).

In addition to the OA, the exudation of other organic compounds in the root has been suggested for the chelation of Al (Kochian et al., 2015); however, very little is known of their mechanism of action. Studies on *Eucalyptus camaldulensis* have revealed secretion in the root of a ligand with low molecular weight binding to Al (Tahara et al., 2008, 2009). In tea plants, an increase in the release of caffeine, a phenolic compound, has been observed in response to exposition to Al (Morita et al., 2011). Other ligands released in the root include the phenolic compounds (catechol, catechin, and quercetin), flavonoids, succinate, phosphates, UDP-glucose and polysaccharides in the form of mucilage (Kidd et al., 2001; Winkel-Shirley, 2002; Zheng et al., 2005; Huang et al., 2009; Osawa et al., 2011; Kochian et al., 2015). In fact, root mucilage plays an important role as a mechanism of resistance to metals (Morel et al., 1986). Mucilage is a gelatinous material consisting of polysaccharides of high molecular weight which are exuded from the most external layers of the root apex. Due to the fact that the mucilage contains uronic acid and pectin, the carboxil groups of this acid and of the pectin can ligate metallic cations such as Al (Watanabe et al., 2008a,b). In cowpea (*Vigna unguiculata*), wheat and corn a strong binding of mucilage-Al has been reported and this bond is not toxic for the plant (Horst et al., 1982; Archambault et al., 1996; Li et al., 2000).

#### CAP Type Structures and Border Cells in the Radical Apex

The function of the root cap or calyptra and the border cells is to protect the mother cells of the root apex from microbes and soil stresses, and also to receive and transmit environmental signals which will ultimately determine root growth (Endo et al., 2011; Kumpf and Nowack, 2015; Hawes et al., 2016). In many plant species, the root cap and border cells produce and exude mucilage, rich in polysaccharides, which can bind to metallic cations (Horst et al., 1982; Cai et al., 2013; Kumpf and Nowack, 2015). In genotypes of bean (*Phaseolus vulgaris*), barley, soybean and castor (*Ricinus communis*), resistant to Al, the exclusion of the metal is associated with the immobilization and detoxification of Al with the mucilage secreted by the root cap and border cells (Miyasaka and Hawes, 2001; Zhu et al., 2003; Cai et al., 2013; Silva et al., 2014). In contrast, the mucilage secreted by the root cap of *M. malabathricum* increases the accumulation of Al in the plant (Watanabe et al., 2008a,b). In pea (*Pisum sativum*) plants, it has been observed that removal of the border cells increases the sensitivity and absorption of Al, indicating the important role of the border cells as mechanisms of Al exclusion (Yu et al., 2009a; Yang J. et al., 2016). Furthermore, in *Acacia mangium*, a legume tropical forest tree known to be resistant to Al, the root apex was found to be surrounded by a cap type structure, the purpose of which is to protect the root from the flexion induced by Al (Endo et al., 2009, 2011).

#### Alleviation of Aluminum-Induced Toxicity

Recent evidence has demonstrated that the exogenous addition and availability of certain elements prevents Al toxicity in plants. In rice, ammonium (NH_4_^+^) reduces the accumulation of Al in the root as a consequence of pH changes induced by NH_4_^+^ uptake and the direct competition of Al and NH_4_^+^ for the cell wall binding sites (Wang et al., 2015d). Similarly, the exogenous addition of Si in corn prevents the inhibition of root elongation and callous deposition through the formation of hydroxy aluminosilicates in the root apex (Wang et al., 2004). In pea and rice, the application of Si reduced the content of Al in the roots, stem and leaves (Singh et al., 2011; Shen et al., 2014). In addition to being cofactor of many enzymes and a central component of chlorophyll, Mg also prevents metal phytotoxicity, including Al. High concentrations of Mg (mM) prevent Al toxicity by competing in uptake and interaction with the binding sites in the cell wall and the plasma membrane (Bose et al., 2011; Rengel et al., 2015). In the cytosol, Mg (μM) can increase the biosynthesis of OA and induce the activity of the H^+^-ATPasa by phosphorylation to increase the resistance to Al (Bose et al., 2013; Rengel et al., 2015). Prevention of Al toxicity by supplementation with B has been reported in a large number of plants. Boron can act synergically with Ca and prevent binding of Al to the cell wall (Hossain et al., 2005; Yu et al., 2009b; Horst et al., 2010). Recent evidence in pomelo (*Citrus grandis*) suggests that B prevents Al-toxicity by regulating different genes associated with modification of the cell wall, cellular transport, metabolism, signal transduction and antioxidant activity (Wang et al., 2015c; Zhou et al., 2015). Similarly, the addition of P prevents the effect of Al-toxicity on root growth and photosynthetic machinery of *C. grandis* (Jianq et al., 2009). The P-deficiency also reduces Al-toxicity by changing the properties of the plasma membrane and cell wall, while high P increases the toxicity of the metal, possibly through the precipitation of Al-P on the root surface (Maejima et al., 2014; Shao et al., 2015).

Hormones and polyamines play an important role in the tolerance to stress by Al. The exogenous addition of auxins (indole-acetic acid, IAA) reduces the accumulation of Al in the root apex in wheat (Wang et al., 2013c) and alleviates the Al-induced inhibition of root growth in lucerne (*Medicago sativa*) (Wang S. et al., 2016). Indole-acetic acid stimulates the exudation of citrate by the positive regulation of the GmMATE transporter and increases the activity of the PM H^+^-ATPasa by phosphorylation. Both processes participate in resistance to stress by Al through chelation of Al-citrate and pH changes in the rhizosphere (Wang S. et al., 2016; Wang P. et al., 2016). Furthermore, the overexpression of a transport auxin efflux (*OsPin2*) prevents rigidity of the cell wall, Al-binding to the cell wall and oxidative damage induced by Al through the transport of auxins and H^+^ (Wu et al., 2014). The polyamine putrescine (Put) and nitric oxide are also involved in the modulation of citrate secretion from roots of red bean (Wang et al., 2013a). The Al-induced root inhibition could be alleviated by Put through decreased ethylene production (Yu Y. et al., 2016). Putrescine has been identified as an important signaling molecule involved in Al tolerance in plants (Chen et al., 2008; Wang et al., 2013b; Yu Y. et al., 2016).

Some factors of abiotic stress such as drought and hypoxia can indirectly reduce the accumulation of Al and its effect on plants. In bean, drought stress changes the porosity of the cell wall and reduces the Al-binding (Zhang et al., 2016); on the other hand, hypoxic stress in barley prevents Al-toxicity in roots by increasing the antioxidant capacity and reducing K efflux induced by ROS (Ma et al., 2016). Mycorrhiza association can also prevent Al-toxicity in acid soils (Seguel et al., 2013).

### Tolerance to Aluminum- Mechanisms of Internal Tolerance

#### Chelation of Aluminum in the Cytosol (Organic Acids and Other Organic Ligands)

The mechanism of internal detoxification of Al involves the chelation of metal with OA and subsequent sequestration into the vacuole. Many tolerant plants, including the hyperaccumulators of Al, use the OA for the sequestration of Al in the cytosol of the root cells and also to remobilize or to translocate Al toward the shoots. *Fagopyrum esculentum* uses oxalate for the internal and external chelation of Al (Ma et al., 1998; Ma and Hiradate, 2000; Shen et al., 2002; Wang et al., 2015a). Oxalate is the predominant ligand in the root cytosol of tea and forms Al-oxalate compounds (Morita et al., 2004); however, Al is translocated toward the shoots in the form of Al-citrate or Al-malate (Morita et al., 2004, 2008). A mechanism similar to that of tea is observed in buckwheat (Wang et al., 2015a). In the shrub *M. malabathricum*, intracellular Al binds to citrate, and the Al-citrate compound is transported to the shoots; once in the leaves, the citrate is substituted by oxalate to form Al-oxalate (1:3) which is less toxic (Watanabe and Osaki, 2001). In *Camellia oleifera* Al is transported via phloem; however, it is not known if it is in the form of an Al-OA compound (Zeng Q.L. et al., 2013).

In addition to the OAs, the phenolic compounds can bind to Al and form a complex in the cytosol. For example, in tea plants, catechin forms a complex with Al (Nagata et al., 1992). In the sepals of *H. macrophylla* Al can bind to chlorogenic acid (3-caffeoylquinic acid) and delphinidin a 3-glucoside for stabilization of Al and color change in the flowers (Ma et al., 1997, 2001). In *C. camphora*, the accumulation of proanthocyanidin in the epidermal cells of the root apex is induced by Al; however, there is no evidence of its binding to metal (Osawa et al., 2011). It has also been suggested that the hydroxamates can bind to Al in the root (Poschenrieder et al., 2005). In the sap of tea leaves, the Al-F compound has been identified as a mechanism of tolerance to F (Yang Y. et al., 2016).

#### Aluminum Transporters in the Plasma Membrane and Vacuolar Compartmentalization

Transportation through biological membranes requires transport proteins. In plants, Al transportation through the plasma membrane and the vacuole tonoplast has not been widely studied. However, it has been reported that the ABC transporters, transporters of binding to ATP, AtABCI16/AtALS3, AtABCI17/AtSATR1/AtALS1 and OsALS1, as well as the transporters Nrat1 (Nramp Family) contribute to the detoxification of Al in plants (Xia et al., 2010; Hwang et al., 2016). AtALS3 is a partial, type ABC transporter which is located in the PM of the epidermal cells of the root cortex, and in phloem cells throughout the plant. It is believed that AtASL3 distributes Al inside the plant far from the root apex (sensitive to Al), by transporting Al directly or bound to a ligand (Kang et al., 2011; Ryan et al., 2011). To date, it is unknown if AtASL3 is a transporter of Al influx or efflux (Delhaize et al., 2012). However, it has been suggested that it might participate in Al efflux from the root after absorption of the metal (Larsen et al., 2005). In fact, the Al efflux has been proposed as an exclusion mechanism (Arunakumara et al., 2013). Mutation of the transporter AtALS3 results in hypersensitivity to Al and an increase in the accumulation of the metal in the roots of *Arabidopsis* (Larsen et al., 1997, 2005). An *ALS3* gene with characteristics similar to those of *AtASL3* has been identified in poplar (*P. tremula*); however, it has been suggested that it participates in internal tolerance of Al (Grisel et al., 2010; Brunner and Sperisen, 2013). AtALS1 is also a partial, type ABC protein and probably participates in intracellular transportation of Al in vacuoles of root cells and vascular cells of the plant (Larsen et al., 2007; Delhaize et al., 2012). *OsALS1* is an ortholog of *AtALS1* in rice and its expression is induced rapidly and specifically by Al. OsALS1 is located in the tonoplast of root cells and participates in the compartmentalization of Al in the vacuoles, which is required for the internal detoxification of Al in rice (Huang et al., 2009, 2012).

The Nrat1 transporter is a member of the Nramp family of transporters located in the PM of all root apex cells, except in the epidermal cells (Xia et al., 2010). The Nrat1 gene is positively regulated by the transcription factor responsive to Al, ART1 in rice (Yamaji et al., 2009). It has been demonstrated that the protein Nrat1 exhibits the activity of Al transportation, but not for divalent metals such as Fe, Mn, and Cd or Al-citrate compounds (Xia et al., 2010, 2011). Moreover, it has been suggested that Nrat1 is required for detoxification of Al in the cell wall as it reduces metal levels through Al-influx to the root cells and their subsequent compartmentalization in the vacuole, possibly by OsALS1 (Xia et al., 2010, 2011, 2014; Huang et al., 2012; Li et al., 2014). Given that the mutant *nrat1* presents increased sensitivity to Al and the over-expression of Nrat1 in yeast, rice and *Arabidopsis* increases Al uptake (Xia et al., 2010, 2011; Li et al., 2014), the Nrat1 gene or its orthologs can be useful tools to enhance Al tolerance in different plant species.

In *H. macrophylla*, transporters have been identified [members of the aquaporin (AQP) family and anion permeases], located in the tonoplast and plasma membrane, which must work as Al transporters (Negishi et al., 2012, 2013). Recently, Wang et al. (2017) reported that NIP1;2, a plasma membrane-localized member of the *Arabidopsis* nodulin 26-like intrinsic protein (NIP) subfamily of the AQP family, facilitates Al-malate transport from the root cell wall into the root symplasm, with subsequent Al xylem loading and root-to-shoot translocation, which are critical steps in an internal Al tolerance mechanism in *A. thaliana*. Surprisingly, NIP1;2 facilitates the transport of Al-malate, but not Al^3+^ ions. Hence the coordinated function of NIP1;2 and ALMT1 are required for Al uptake, translocation, and tolerance in *Arabidopsis* (Hoekenga et al., 2006; Wang et al., 2017). Moreover, a recent report mentions that endocytic vesicular traffic can contribute to the internalization of Al in the root apex of rice. The overexpression of *OsPIN2* in rice increases tolerance to Al through the internalization of Al via vesicular traffic (Wu et al., 2015).

#### Modification of Plant Metabolism and DNA Checkpoints

In plants, the mechanisms of exclusion and tolerance to Al are intimately related to mitochondrial activity, mitochondrial metabolism and OA transportation (Nunes-Nesi et al., 2014). The cycle of TCA is inducible by Al, and intervenes in the biosynthesis of OA to chelate Al in the apoplast or cytosol. In *Brassica napus*, Al induces enzyme activity of OA anabolism and catabolism, as well as the accumulation of OA (Ligaba et al., 2004). Overexpression of the enzymes citrate synthase, malate dehydrogenase and pyruvate phosphate dikinase confers resistance to Al by increasing the synthesis and exudation of OA (Deng et al., 2009; Han et al., 2009; Trejo-Téllez et al., 2010; Wang et al., 2010). In wheat varieties tolerant to Al, exposure to Al positively regulates the expression of mitochondrial ATP synthase and vacuolar H^+^-ATPasa, suggesting an increase in metabolic activity in the mitochondria and pH changes in the cytosol (Hamilton et al., 2001). Therefore, the increase in mitochondrial biochemical activity is important for the synthesis of OA under stress by Al. In fact, the lack of correlation between OA exudation and resistance to Al in corn, soybean and buckwheat (Piñeros et al., 2005; Nian et al., 2005; Zheng et al., 2005) has suggested that the exudation of OA is a result of the biochemical reactions required for the tolerance to Al (Nunes-Nesi et al., 2014).

Alternative metabolic routes of cellular respiration also participate in tolerance to stress by Al. Under adverse conditions, where carbohydrates are scarce, plants can metabolize proteins and lipids as alternative substrates for cellular respiration (Araújo et al., 2011). Similarly, the overexpression of mitochondrial alternative oxidase (AOX) increases the respiration and reduces the oxidative stress induced by Al in the mitochondria (Panda et al., 2013). Lou et al. (2016) suggested that formate accumulation is involved in both H^+^ and Al-induced root growth inhibition in rice bean (*Vigna umbellate*). The overexpression of the formate dehydrogenase (*FDH*) gene of rice bean (*VuFDH*) in tobacco (*N. tabacum*) results in decreased sensitivity to Al and H^+^ stress due to less production of format in the transgenic tobacco lines under Al and H^+^ stresses. These findings suggest a possible new route toward the improvement of plant performance in acidic soils, where Al toxicity and H^+^ stress coexist (Lou et al., 2016). In tomato (*Solanum lycopersicum*) roots proteins have been identified which play an important role in Al exclusion and tolerance (Zhou S. et al., 2016). Gallagher et al. (1980) evaluated nitrate reductase activity and reported an increase in the activity of this enzyme in crops which are tolerant to Al. Toxicity by Al is also associated with the metabolism of nitrogen (N) (Foy and Fleming, 1982). Transcriptome analyses in lucerne roots reveal candidate Al-stress-responsive genes involved in ribosome, protein biosynthesis, TCA cycle, membrane transport (organic, small molecules and ions) and hormonal regulation. However, the ribosome protein genes was the pathway with the largest numbers of genes differentially up-regulated, which suggested a high biological importance for ribosomal genes and an alternative in response to Al stress in plants (Liu et al., 2017). Also, NAC genes have been postulated to play pivotal roles in plants exposed to Al (Moreno-Alvarado et al., 2017).

Recently identified *Arabidopsis* mutants with increased Al tolerance provide evidence of DNA as one of the main targets of Al (Eekhout et al., 2017). Al treatment results in binding of Al to the negative charges of the phosphodiester backbone DNA. In fact, nuclei have been reported to accumulate Al even in the presence of low environmental concentrations. Binding Al to DNA might possibly alter DNA topology from the B-DNA to Z-DNA conformation, resulting in increased DNA rigidity that leads to difficulty in unwinding during DNA replication and susceptibility of DNA to endogenous mutagens (Anitha and Rao, 2002; Hu et al., 2016). Also, Al stress gives rise to changes in the localization and expression of the nucleolar proteins and inhibition of DNA synthesis, as well as promoting DNA fragmentation and the generation of micronuclei (Zhang et al., 2014; Sade et al., 2016; Eekhout et al., 2017). In *Arabidopsis* plants, this Al- induced DNA damage triggered the activation of a cell cycle arrest causing root growth inhibition, at least partly (Rounds and Larsen, 2008). Eekhout et al. (2017) suggested the modification of DNA checkpoints to confer Al-tolerance through DNA repair with cell-cycle progression. The DNA damage response (DDR) pathway maintains genome integrity under adverse conditions that affect DNA replication. The DDR pathway introduces a transient cell-cycle arrest during the process of DNA repair, through the coordinated expression of DNA repair and cell-cycle inhibitory genes, thus ensuring that both the daughter cells inherit a complete and error free copy of the genome. This insight could lead the way for novel strategies to generate Al-tolerant crop plants (Hu et al., 2016; Eekhout et al., 2017).

## Conclusion

Much interest has been shown recently in the use of biostimulants and stimulants in agriculture with the aim of increasing root growth, nutrient uptake and tolerance to stress in plants. Plant biostimulants or agricultural stimulants include microorganisms and a diversity of substances, among which are the beneficial elements (Calvo et al., 2014). Aluminum (Al), cobalt (Co), selenium (Se), sodium (Na), and silicon (Si) are considered to be beneficial elements for plants, given that, despite the fact that they are not required by all plants, they can promote growth and are essential for certain plant taxa, depending on the environmental conditions, concentration of the element and plant species. These elements can also increase tolerance to abiotic stress (drought, salinity, high temperatures, cold, UV light, toxicity or nutrient deficiency) as well as biotic stress (pathogens and herbivores) when administered at low concentrations. However, it is important to know the range of concentration in which a beneficial element becomes lethal, in particular with respect to its use as a fertilizer to increase the production of crops under conditions of stress and/or improve the nutritional value of food plants (Pilon-Smits et al., 2009; Kaur et al., 2016).

Aluminum stimulates growth in plants of economic importance such as the tea shrub and can maintain or fix floral colors, as in the case of hydrangeas; therefore its application as a biostimulant of a desirable response in these plants is feasible (Fang et al., 2014). However, high concentrations of Al can pose a serious threat to agricultural production due to inhibition of root elongation and plant growth through a diversity of mechanisms with the participation of Al, including interactions in the symplast, plasma membrane and the cell wall (Kochian et al., 2015). In addition to the plants, Al can also cause serious problems in the nervous system, lungs and kidneys of human beings. Tea is an important dietetic source of Al for human beings. There is a great need to understand how environmental factors can have an influence on the accumulation of Al in tea leaves in order to create strategies for the reduction of Al uptake in tea plants (de Silva et al., 2016). A number of studies have suggested that people who are exposed to high levels of Al can develop Alzheimer’s disease, encephalopathy and dementia, among other diseases (Anitha and Rao, 2002; Mehra and Baker, 2007; Ashenef, 2014).

The root system is complex and a wide variety of root phenotypes have been identified which contribute to the adaptation to toxicity by Al (Rao et al., 2016). A greater root surface induced by Al can increase the uptake of water and nutrients by plants (Hajiboland et al., 2013b), mainly in conditions of stress caused by drought, salinity and nutrient deficiency. Further studies on the use of low concentrations of Al to prevent the effect of different conditions of stress must be considered.

## Author Contributions

EB-Q designed, planned, wrote, and checked the manuscript. CE-M wrote the manuscript. IE-M checked the manuscript. MM-E group leader, wrote and checked the manuscript.

## Conflict of Interest Statement

The authors declare that the research was conducted in the absence of any commercial or financial relationships that could be construed as a potential conflict of interest.
